# *Listeria monocytogenes* Growth Kinetics in Milkshakes Made from Naturally and Artificially Contaminated Ice Cream

**DOI:** 10.3389/fmicb.2018.00062

**Published:** 2018-01-24

**Authors:** Joelle K. Salazar, Vriddi M. Bathija, Christina K. Carstens, Sartaj S. Narula, Arlette Shazer, Diana Stewart, Mary Lou Tortorello

**Affiliations:** ^1^Division of Food Processing Science and Technology, U.S. Food and Drug Administration, Bedford Park, IL, United States; ^2^Institute for Food Safety and Health, Illinois Institute of Technology, Bedford Park, IL, United States

**Keywords:** ice cream, *Listeria monocytogenes*, growth kinetics, growth model, natural contamination

## Abstract

This study assessed the growth of *Listeria monocytogenes* in milkshakes made using the process-contaminated ice cream associated with a listeriosis outbreak in comparison to milkshakes made with artificially contaminated ice cream. For all temperatures, growth kinetics including growth rates, lag phases, maximum populations, and population increases were determined for the naturally and artificially derived contaminants at 5, 10, 15, and 25°C storage for 144 h. The artificially inoculated *L. monocytogenes* presented lower growth rates and shorter lag phases than the naturally contaminated populations at all temperatures except for 5°C, where the reverse was observed. At 25°C, lag phases of the naturally and artificially contaminated *L. monocytogenes* were 11.6 and 7.8 h, respectively. The highest increase in population was observed for the artificially inoculated pathogen at 15°C after 96 h (6.16 log CFU/mL) of storage. Growth models for both contamination states in milkshakes were determined. In addition, this study evaluated the antimicrobial effectiveness of flavoring agents, including strawberry, chocolate and mint, on the growth of the pathogen in milkshakes during 10°C storage. All flavor additions resulted in decreased growth rates of *L. monocytogenes* for both contamination states. The addition of chocolate and mint flavoring also resulted in significantly longer lag phases for both contamination states. This study provides insight into the differences in growth between naturally and artificially contaminated *L. monocytogenes* in a food product.

## Introduction

*Listeria monocytogenes* is commonly present in bulk tank milk (Jackson et al., 2012) and has long been recognized to be able to survive in facilities where dairy products are processed, including frozen dairy products such as ice cream. A listeriosis outbreak, which occurred in the United States between the years 2010 and 2015 and resulted in 10 total cases, 100% hospitalization rate, and three deaths, was linked to the consumption of ice cream products, which became contaminated post-pasteurization by *L. monocytogenes* present in the processing environment (CDC, 2015; FDA, 2015). Four of the cases resulted from consuming milkshakes prepared from the contaminated ice cream at a healthcare facility. The ice cream was packaged into single-serving units, and it has been determined that approximately 92% of these samples were contaminated with at least two strains of *L. monocytogenes* at a nearly uniform level of less than 20 most probable number (MPN)/g (Chen, 2015; Chen et al., 2016b, 2017).

The availability of uniformly and naturally contaminated samples of the ice cream product provided an unusual opportunity to study the behavior of *L. monocytogenes* contaminants in a realistic condition. Challenge studies are generally conducted using artificial inoculation of food products with a target pathogen. Naturally contaminated food products are rarely available for research purposes because they are often destroyed when a recall or outbreak occurs. However, a few published reports do exist in which the behavior of *L. monocytogenes* was assessed in naturally contaminated foods, and these included smoked salmon (Cortesi et al., 1997; Dalgaard and Jørgensen, 1998; Lappi et al., 2004; Beaufort et al., 2007), seafood (Jørgensen and Huss, 1998; Mejlholm et al., 2015), chorizo (Encinas et al., 1999) and raw meat (Farber and Daley, 1994; Ryser et al., 1996). Only one study involved a naturally contaminated dairy product, i.e., the ice cream associated with the 2010–2015 listeriosis outbreak (Chen et al., 2016a).

It is not clear how pathogen growth and survival data might differ between artificial and natural contaminants when used in challenge studies. Any differences could be usefully considered if the data were to be used for risk assessment purposes. In this study, milkshakes were made using both naturally and artificially contaminated ice cream products to determine whether the behavior of the pathogen would differ between the two modes of contamination. Storage temperatures of 5, 10, 15, and 25°C were chosen to simulate a range of temperature holding and abuse conditions. For example, in the case of the recent outbreak, milkshakes might have been previously made and then stored under refrigeration (5°C) before being given to the patients; or they might have been left at room temperature (25°C) and consumed at a later time. The inclusion of additional temperatures would allow a better understanding of the influence of temperature on the growth of both naturally and artificially contaminated *L. monocytogenes*. In addition, this study also aimed to determine the effect of flavoring agents, which have demonstrated antimicrobial activity (Puupponen-Pimiä et al., 2005; Nohynek et al., 2006; Lixandru et al., 2010; Akdemir Evrendilek, 2015), on the growth and survival of *L. monocytogenes* in the milkshakes. The comparison of *L. monocytogenes* behavior in flavored and unflavored milkshakes, made with naturally or artificially contaminated ice cream samples and storage at various temperatures, could help to determine the adequacy of laboratory studies conducted with artificially inoculated food samples for use in risk assessment via predictive modeling.

## Materials and Methods

### *L. monocytogenes* Strains and Culture Conditions

Resistance to rifampicin (100 μg/mL) was selected in five *L. monocytogenes* strains: F2365 (serotype 4b, isolated from Mexican soft cheese) (Linnan et al., 1988; Chen et al., 2016c), ScottA (serotype 4b, sequence type 290, isolated from milk) (Fleming et al., 1985; Briers et al., 2011), FSL R2-502 (serotype 1/2b, sequence type 3, isolated from chocolate milk) (Dalton et al., 1997; Chen et al., 2016c), LS806 (serotype 4b, sequence type 1, isolated from cheese), and JKS-1, a strain isolated from the process-contaminated ice cream associated with the listeriosis outbreak. The naturally contaminated *L. monocytogenes* found in the ice cream (including JKS-1) belong to sequence type 5, molecular serogroup IIb, and genetic lineage I (Chen et al., 2017). All strains were grown separately in Brain Heart Infusion (BHI, Becton Dickinson & Co., Sparks, MD, United States) broth containing rifampicin at 37°C for 16–18 h with 150 rpm shaking. A cocktail of F2365, Scott A, R2-502, and LS806 or JKS-1 by itself was used for artificial inoculation of ice cream.

### Artificial Inoculation of Naturally Contaminated Ice Cream

**FIGURE 1:**
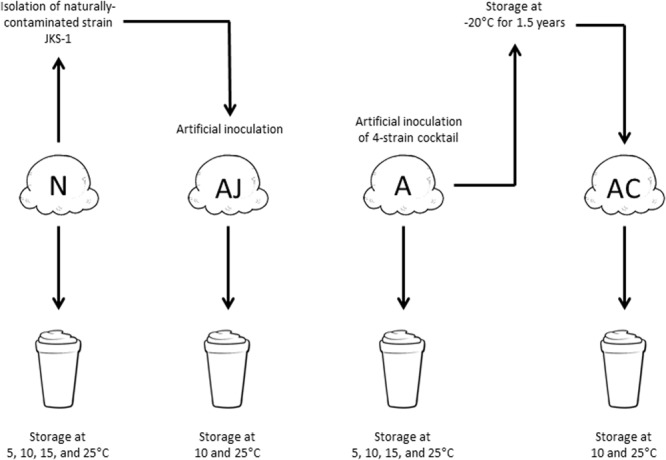
The four contamination situations of *L. monocytogenes* utilized in this study. N, naturally contaminated; AJ, artificially contaminated JKS-1; A, artificially contaminated; AC, artificially contaminated 1.5-year-cold-adapted.

The *L. monocytogenes* cultures were washed twice with Butterfield’s phosphate buffer (BPB), pH 7.4, combined if used as a cocktail, and diluted to 4 log CFU/mL. Individual 80-g ice cream product samples (“scoops”), which were naturally contaminated with approximately 10 MPN/g *L. monocytogenes* during manufacture (Chen, 2015), were divided into four groups (**Figure 1**): one group remained as naturally contaminated (N), the second group was artificially contaminated with the four-strain cocktail (A), and the third group was artificially contaminated with JKS-1 (AJ). For groups A and AJ, 80 μL of the cocktail or single strain was pipetted into the center of each ice cream scoop with a wide-orifice pipette tip, resulting in a final concentration of 10 CFU/g. A subset of the artificially contaminated ice cream samples (A) was stored at -20°C for 1.5 years (AC; cold-adapted artificially inoculated).

### Preparation of Milkshakes

Milkshakes were prepared according to the recipe used at the outbreak-associated healthcare facility (Yi Chen, personal communication). Individual ice cream scoops (N, A, AJ, or AC; see **Figure 1**) were placed into sterile metal mixing cups and combined with 60 mL ultra-high temperature pasteurized (UHT) 1% milk. UHT milk was used to decrease background microbiota on agar plates. For milkshakes with flavoring agents (N and A; see **Figure 1**), commercial strawberry syrup (15 mL), chocolate syrup (15 mL), or mint extract (2.5 mL) was added. Samples were homogenized for 2 min at medium speed using a commercial Hamilton Beach HMD200 Drink Mixer (Hamilton Beach, Inc., Southern Pines, NC, United States) and then were transferred to 125 mL sterile polystyrene cups with lids. Milkshakes were stored at 5, 10, 15, and 25°C (for A and N; see **Figure 1**) or at 10 and 25°C (for AJ and AC; see **Figure 1**) for 12, 24, 48, 72, 96, and 144 h. For 25°C, additional timepoints of 3, 6, and 9 h were included. Four milkshake replicates were assessed for each group at each timepoint. Three independent experiments were conducted for A and N milkshakes and two independent experiments were conducted for AJ and AC milkshakes.

### Enumeration of Naturally and Artificially Contaminated *L. monocytogenes* from Milkshakes

At each timepoint, duplicate 40-g quantities of milkshake were each placed into a 1-L stomacher bag with 450 mL Buffered *Listeria* Enrichment Broth (BLEB, Becton Dickinson & Co., Sparks, MD, United States). For artificially inoculated samples (A, AJ, and AC milkshakes), rifampicin was added to the BLEB to a final concentration of 100 μg/mL to eliminate the population of natural *L. monocytogenes* contaminants. Samples were stomached for 1 min at 180 rpm in a Seward 3500 stomacher (Seward Laboratory Systems Inc., Davie, FL, United States). The FDA Most Probable Number (MPN) method (Hitchins et al., 2016) was used to enumerate the low levels of *L. monocytogenes*. The MPN scheme included four levels of dilutions: 3 bags of 100 mL each, 5 tubes of 10 mL each, 8 tubes of 1 mL each, and 8 tubes of 0.1 mL each (Chen, 2015), resulting in final milkshake sample quantities of 8.2, 0.82, 0.082, and 0.0082 mL, respectively. The MPN bags and tubes were incubated at 30°C, with the addition of BLEB supplement (Oxoid Ltd., Hampshire, United Kingdom) after 4 h of incubation. After 24 and 48 h of incubation, all samples were streaked onto Brilliance *Listeria* agar (Oxoid) for determining the natural *L. monocytogenes* (N) and onto Plate Count Agar (Becton Dickinson & Co., Sparks, MD, United States) supplemented with 100 μg/mL rifampicin for determining the artificial *L. monocytogenes* (A, AJ, and AC). *L. monocytogenes* MPN concentrations were calculated using the FDA Excel (Microsoft, Redmond, WA, United States) spreadsheet freely available at http://www.fda.gov/Food/FoodScienceResearch/LaboratoryMethods/ucm109656.htm. At timepoints where *L. monocytogenes* was above the limit of detection of the MPN scheme (approximately 253 MPN/mL), the stomached samples were serially diluted and directly plated. All agar plates were incubated at 37°C for 48 h.

### Predictive Primary and Secondary Modeling

The DMFit version 3.0 (Institute of Food Research, Norwich, United Kingdom) Excel add-on from ComBase ^1^ was used to model the maximum growth rates (μ_max;_ log CFU/mL/h), lag phases (λ; h), and maximum population densities (*y*_max_; log CFU/mL) of the naturally and artificially contaminated *L. monocytogenes* at each temperature, with or without flavoring addition, using the Baranyi model (Baranyi and Roberts, 1994). Secondary modeling of growth rate was determined using the Ratkowsky square root equation (eq. 1) (Ratkowsky et al., 1982). From the equation, a comparison of lag time with temperature can be determined (eq. 2). Parameters *b* and *T*_min_ (°C) were computed for each model.

(1) $$\begin{array}{c} \sqrt{\;\mu_{max}}=b\left(T-T_{min}\right) \end{array}$$

(2) $$\begin{array}{c} \ln\left(\;\lambda \right)=\ln\left[\frac{1}{{\left(b\left(T-T_{min}\right)\right)}^{2}}\right] \end{array}$$

where *b* is the regression coefficient, *T* is temperature, and *T*_min_ is the theoretical minimum temperature below which microbial growth does not occur.

### Statistical Analysis

Statistical analyses were evaluated using GraphPad Prism v 7.0 and GraphPad InStat v 3.0 (GraphPad Software, Inc., La Jolla, CA, United States). Tukey’s adjusted one-way analysis of variance was utilized and a *P* value less than 0.05 was considered significant.

## Results

### Growth Kinetics of *L. monocytogenes* in Milkshakes Made from Naturally and Artificially Contaminated Ice Cream

**FIGURE 2:**
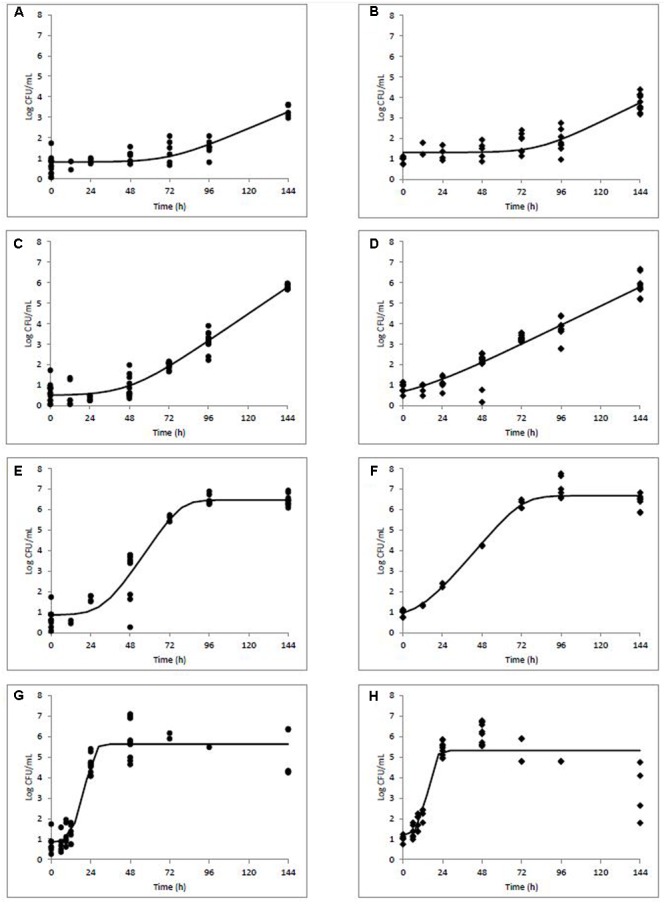
Modeled growth of *L. monocytogenes* in milkshakes made from naturally and artificially contaminated ice cream stored at 5 **(A,B)**, 10 **(C,D)**, 15 **(E,F)**, or 25°C **(G,H)** for 144 h. Data points represent CFU per mL of milkshake. Line represents modeled growth. Circles **(A,C,E,G)** are naturally contaminated and diamonds **(B,D,F,H)** are artificially contaminated *L. monocytogenes* populations.

Growth rates, lag phases, and maximum population densities were determined based on the model of Baranyi and Roberts (1994) using DMFit for both naturally and artificially contaminated *L. monocytogenes* in milkshakes during 144 h storage at four temperatures (**Table 1**). Maximum population’s densities were also determined for both contamination states at all temperatures. **Figure 2** depicts growth of the natural and artificial *L. monocytogenes* contamination states (N and A, see **Figure 1**) in milkshakes during storage at 5 (**Figures 2A,B**), 10 (**Figures 2C,D**), 15 (**Figures 2E,F**), and 25°C (**Figures 2G,H**). At 5°C, no significant differences were observed between the growth rates or lag phases of the naturally or artificially contaminated *L. monocytogenes*. An overall increase in population of approximately 2.58 and 2.79 log CFU/mL was seen for the natural and artificial contamination states, respectively, after 144 h in milkshakes stored at 5°C. At 10, 15, and 25°C, the growth rates of the naturally contaminated *L. monocytogenes* were significantly higher than those of the artificially contaminated. The lag phases of the naturally contaminated pathogen were also significantly longer than the artificially contaminated at 10, 15, and 25°C. However, the maximum attained populations of either state were not significantly different. At 10°C, overall increases in populations of approximately 5.15 and 5.04 log CFU/mL were observed for the natural and artificial contamination states, respectively, after 144 h storage. The highest population increases, approximately 5.93 and 6.16 log CFU/mL, were observed by the naturally and artificially contaminated pathogen at 15°C after only 96 h storage. The shortest lag phases overall at approximately 12 and 8 h, were observed by the naturally and artificially contaminated *L. monocytogenes* at 25°C, respectively.

**Table 1:** Growth kinetics of *L. monocytogenes* in milkshakes (A and N, see **Figure 1**) made from naturally and artificially contaminated ice cream and stored at different temperatures.

Temp (°C)	*L. monocytogenes* condition	Initial population^∗^, (log CFU/mL ±*SD*)	Growth rate, μ_max_ (log CFU/mL/h ±*SD*)	Lag phase, λ (h ±*SD*)	Maximum population, *y*_max_ (log CFU/mL ±*SD*)	*r*^2^
5	Natural	0.54 ± 0.34	0.034 ± 0.006^a^	71.8 ± 11.1^a^	3.12 ± 0.12^a^	0.77
	Artificial	0.99 ± 0.17	0.039 ± 0.006^a^	82.1 ± 10.0^a^	3.78 ± 0.35^a^	0.84
10	Natural	0.64 ± 0.48	0.055 ± 0.002^a^	47.4 ± 3.5^a^	5.79 ± 0.11^a^	0.89
	Artificial	0.88 ± 0.22	0.039 ± 0.003^b^	12.5 ± 7.9^b^	5.92 ± 0.76^a^	0.93
15	Natural	0.54 ± 0.34	0.118 ± 0.022^a^	31.5 ± 5.4^a^	6.47 ± 0.23^a^	0.94
	Artificial	0.99 ± 0.17	0.094 ± 0.015^b^	11.7 ± 7.2^b^	7.15 ± 0.64^a^	0.98
25	Natural	0.61 ± 0.56	0.303 ± 0.053^a^	11.6 ± 2.3^a^	6.05 ± 0.13^a^	0.93
	Artificial	1.12 ± 0.18	0.270 ± 0.022^b^	7.8 ± 1.4^b^	6.16 ± 0.51^a^	0.98

*^∗^Initial population refers to enumerated pathogen contamination immediately after mixing of milkshakes. SD, standard deviation. No significant differences between initial populations were observed. Different lowercase letters indicate significant difference (*P* < 0.05) between specific parameters for natural and artificial *L. monocytogenes* contamination in milkshakes stored at the same temperature.*

For comparison, the naturally contaminating *L. monocytogenes* strain JKS-1, which was isolated from the ice cream, was artificially inoculated into the ice cream samples (AJ) for growth kinetics experiments in milkshakes. Results indicated that the 10 and 25°C growth rates of the four-strain cocktail (A; 0.039 ± 0.01 and 0.270 ± 0.02 log CFU/mL per h, respectively, see **Table 1**) and the JKS-1 naturally contaminating strain (AJ; 0.042 ± 0.01 and 0.275 ± 0.05 log CFU/mL per h, respectively) were not significantly different at each temperature. In addition, ice cream with 1.5-year-cold-adapted artificially contaminated *L. monocytogenes* (AC) was used to prepare milkshakes. The growth rates of the cold-adapted *L. monocytogenes* (AC) at 10 and 25°C were 0.035 ± 0.01 and 0.213 ± 0.09 log CFU/mL per h, respectively. These growth rates were also not significantly different than those of the artificially inoculated *L. monocytogenes* (A) which was not cold-adapted.

### Effect of Flavoring Agents on the Growth of *L. monocytogenes* in Naturally and Artificially Contaminated Ice Cream Milkshakes

**FIGURE 3:**
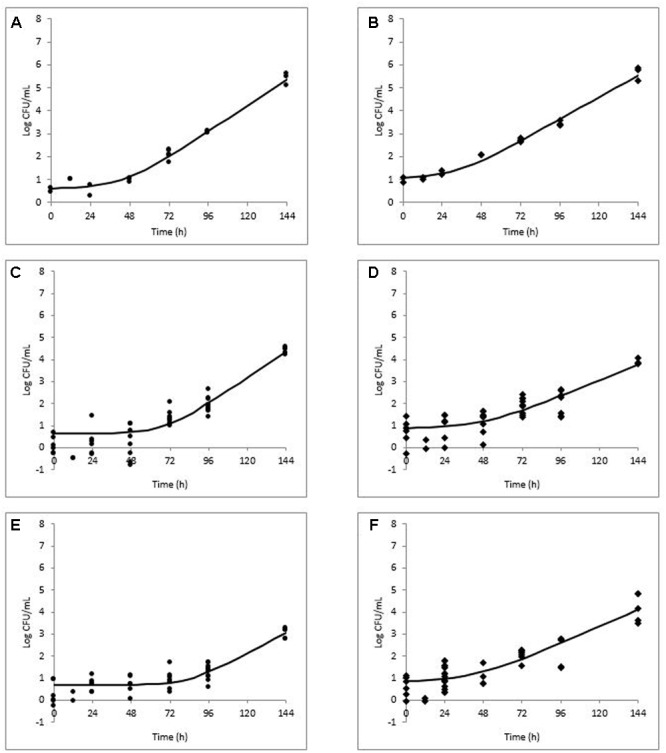
Modeled growth of *L. monocytogenes* in milkshakes made from naturally and artificially contaminated ice cream with the addition of strawberry **(A,B)**, chocolate **(C,D)**, or mint **(E,F)** flavorings during storage at 10°C for 144 h. Data points represent CFU per mL of milkshake. Line represents modeled growth. Circles **(A,C,E)** are naturally contaminated and diamonds **(B,D,F)** are artificially contaminated *L. monocytogenes* populations.

Growth kinetics parameters as determined in the flavoring study are listed in **Table 2**. All growth data for *L. monocytogenes* during storage at 10°C in milkshakes (A and N, see **Figure 1**) with strawberry (**Figures 3A,B**), chocolate (**Figures 3C,D**), and mint flavorings are shown in **Figures 3E,F**.

**Table 2:** Growth kinetics of *L. monocytogenes* at 10°C in milkshakes (A and N, see **Figure 1**) made from naturally and artificially contaminated ice cream and with different flavoring agents.

Flavor	*L. monocytogenes* condition	Initial population^∗^, (log CFU/mL ±*SD*)	Growth rate, μ_max_ (log CFU/mL/h ±*SD*)	Lag phase, λ (h ±*SD*)	Maximum population, *y*_max_ (log CFU/mL ±*SD*)	*r*^2^
None^#^	Natural	0.64 ± 0.48	0.055 ± 0.002^a^^a^	47.4 ± 3.5^a^	5.79 ± 0.11^a^	0.89
	Artificial	0.88 ± 0.22	0.039 ± 0.003^b^^a^	12.5 ± 7.9^b^	5.92 ± 0.76^a^	0.93
Strawberry	Natural	0.55 ± 0.53	0.044 ± 0.005^a^^b^	41.4 ± 5.4^a^	5.55 ± 0.07^a^	0.95
	Artificial	0.88 ± 0.15	0.029 ± 0.002^b^B	10.4 ± 5.0^b^	5.82 ± 0.05^b^	0.99
Chocolate	Natural	0.67 ± 0.84	0.049 ± 0.004^a^^b^	68.2 ± 5.3^a^	4.42 ± 0.14^a^	0.92
	Artificial	0.86 ± 0.57	0.030 ± 0.005^b^B	47.7 ± 13.9^a^	3.90 ± 0.13^b^	0.76
Mint	Natural	1.14 ± 0.17	0.038 ± 0.005^a^^b^	83.0 ± 7.0^a^	3.06 ± 0.23^a^	0.84
	Artificial	0.66 ± 0.56	0.036 ± 0.006^a^A	54.2 ± 12.4^b^	4.19 ± 0.65^b^	0.77

*^∗^Initial population refers to enumerated pathogen contamination immediately after mixing of milkshakes. ^#^The 10°C storage data from **Table 1** are repeated here for convenient comparison. SD, standard deviation. No significant differences between initial populations were observed. Different lowercase letters indicate significant difference (*P* < 0.05) between specific parameters for natural and artificial *L. monocytogenes* contamination in milkshakes made with the same flavoring agent. Different uppercase letters indicate significant difference (*P* < 0.05) between growth rates of naturally or artificially contaminated *L. monocytogenes* in milkshakes made with different flavoring agents.*

In milkshakes flavored with strawberry (N and A milkshakes), the naturally contaminated *L. monocytogenes* population had a significantly higher growth rate, yet significantly longer lag phase than its artificial counterpart, similar to what was observed in milkshakes without flavoring. No significant difference was determined between maximum attained populations by either pathogen contamination state in milkshakes with strawberry flavoring after 144 h. An overall increase of apapproximately 4.88 and 4.94 log CFU/mL in populations of the natural and artificial contamination states was observed in milkshakes with strawberry flavoring, respectively.

In milkshakes supplemented with chocolate flavoring, the naturally contaminated *L. monocytogenes* had a significantly higher growth rate and maximum attained population after 144 h than the artificially contaminated condition. Lag phases of the pathogen in milkshakes with chocolate flavoring increased by approximately 21 and 35 h as compared with milkshakes without flavoring for the naturally and artificially contaminated conditions, respectively. An overall increase in population of only approximately 3.75 and 3.04 log CFU/mL was observed by natural and artificial contamination states, respectively, after 144 h storage in milkshakes with chocolate flavoring.

In milkshakes with mint flavoring, the lowest growth rate was observed for the naturally contaminated *L. monocytogenes*; both contamination states also had the longest lag phases in mint-flavored milkshakes, approximately 83 and 54 h for the naturally and artificially contaminated *L. monocytogenes*, respectively. After 144 h, approximately 1.92 and 3.53 log CFU/mL increases in population were observed for the naturally and artificially contaminated *L. monocytogenes*, respectively, in milkshakes with mint flavoring.

Overall, the growth rates of both the naturally and artificially contaminated *L. monocytogenes* were significantly lower in milkshakes made with the tested flavoring agents than in those without flavoring. The greatest effect of the flavoring agents on *L. monocytogenes*, when taking into account all three kinetic parameters (growth rate, lag phase, and maximum population), was observed by mint, followed by chocolate, whereas the least effect was seen by strawberry. Regardless of flavoring addition or no addition, the naturally contaminated *L. monocytogenes* had higher growth rates and longer lag phases than the artificial counterpart.

### Secondary Modeling of Growth Rate and Lag Phase with Temperature

**FIGURE 4:**
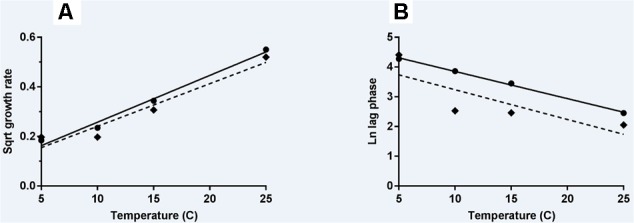
Secondary models of relationship of temperature with growth rate **(A)** and lag phase duration **(B)** of *L. monocytogenes*. Solid line and circles represent naturally contaminated model and observed values, respectively. Dashed line and diamonds represent artificially contaminated model and observed values, respectively.

The effect of storage temperature with *L. monocytogenes* growth rates and lag phases was modeled using the Ratkowsky equation (Ratkowsky et al., 1982) for N and A milkshakes (**Figures 4A,B**, respectively). Parameters and statistical analyses are presented in **Table 3**. Both derived models for comparing growth rate with temperature of the naturally and artificially contaminated *L. monocytogenes* in milkshakes presented with high *r*^2^ values of 0.985 and 0.934, respectively. The derived model for comparing lag phase with temperature for the naturally contaminated *L. monocytogenes* in milkshakes presented a high *r*^2^ value of 0.997. However, the model for the artificially contaminated *L. monocytogenes* in milkshakes fit the Ratkowsky-type model poorly (*r*^2^ = 0.658) due to insignificant differences in lag phases when stored at 10 and 15°C, indicating that the Ratkowsky-type model may not be the most accurate when assessing artificial-contamination.

**Table 3:** Parameters and statistical analyses of the derived secondary models for growth rate and lag phase for *L. monocytogenes* in milkshakes (A and N, see **Figure 1**) made with naturally and artificially contaminated ice cream.

Kinetic parameter	*L. monocytogenes* condition	Model parameter	Estimated value ±*SE*	*P*	*r*^2^
μ_max_	Natural	*b*	0.019 ± 0.002	0.007	0.986
		*T*_min_ (°C)	–3.638		
	Artificial	*b*	0.017 ± 0.003	0.033	0.934
		*T*_min_ (°C)	–4.01		
λ	Natural	*b*	–0.091 ± 0.003	0.001	0.997
		*T*_min_ (°C)	0.019		
	Artificial	*b*	–0.101 ± 0.06	0.189	0.658
		*T*_min_ (°C)	0.024		

*b, regression coefficient. *T*_*min*_ (°C), minimum extrapolated theoretical temperature for growth. CI, confidence interval. ND, not determined. SE, standard error.*

## Discussion

This study assessed the growth dynamics of *L. monocytogenes* in milkshakes made from both artificially and naturally contaminated ice cream. A comparison of the behavior of natural vs. artificial contaminants in a food product has not been reported in the published literature. The naturally contaminated ice cream utilized in this research was uniformly contaminated with 10 MPN/g of *L. monocytogenes* (Chen, 2015); therefore, this inoculation level was used to inoculate artificially contaminated samples for experiments in this study to simulate a real-world situation.

For *L. monocytogenes* growth kinetics comparisons, four different contamination situations were assessed: naturally contaminated (N), artificially contaminated (A, 4 strain cocktail), artificially contaminated (AJ, JKS-1 naturally contaminated strain), and 1.5-year cold adaptation of the artificially contaminated 4 strain cocktail (AC). Since the growth models developed in this study utilized N and A milkshakes, it was important to determine if the growth kinetics of *L. monocytogenes* would change under different situations (AJ and AC). When growth rates of AJ and AC *L. monocytogenes* were compared against those of A at 10 and 25°C (**Table 1**), no differences were observed. This finding therefore reinforces the growth models developed in this study for *L. monocytogenes* in a milkshake matrix.

Research assessing the growth of *L. monocytogenes* in milkshakes made using the same naturally contaminated ice cream used in this study was conducted recently by Chen et al. (2016a). In their study, growth kinetics were compared in milkshakes made from the naturally contaminated ice cream with the addition of strawberry flavoring, chocolate flavoring, or no added flavoring. The milkshakes were stored at room temperature for 14 h, with hourly sampling following a blending step. Results indicated that lag phases of the naturally contaminated *L. monocytogenes* were not significantly different for the strawberry, chocolate, or no added flavoring conditions. The current study utilized a much longer storage time (144 h) in order to determine predictive growth models for both the natural and artificial contaminant for risk assessment purposes. The current study also assessed a slightly higher concentration of flavoring in the milkshakes and also used UHT milk (see section Materials and Methods). These dissimilarities in experimental protocol could provide an explanation as to why significant differences in lag phases were observed in the current study between milkshakes with different flavorings.

Other similar studies have assessed *L. monocytogenes* growth in various dairy products although not in milkshakes. One study determined the growth kinetics of artificially inoculated *L. monocytogenes* in ice cream during storage at various cold and freezing temperatures (Gougouli et al., 2008). The study determined that growth rate of *L. monocytogenes* was significantly higher at 16°C, as compared to 4°C storage, and that the maximum population density was not affected by storage temperature. The study also modeled the effect of temperature (4, 8, 12, and 16°C) on the growth rate of the pathogen in the ice cream samples using the Ratkowsky equation (Ratkowsky et al., 1982) and found *b* and *T*_min_ parameters to be 0.0203 and -2.102, respectively, similar to what was determined in the current study (**Table 3**) and other published studies (Alavi et al., 1999; Augustin et al., 2005; Xanthiakos et al., 2006). Another study which assessed the growth of artificially inoculated *L. monocytogenes* in pasteurized vanilla cream determined growth kinetics of the pathogen at storage temperatures of 3, 5, 10, and 15°C (Panagou and Nychas, 2008). *b* and *T*_min_ parameters were determined to be 0.017 and -6.30, respectively, and these values are also similar to what was found in the current study.

Strawberry, chocolate, and mint flavorings were used in milkshakes in this study to determine if the addition of flavoring agents hindered the growth of the naturally or artificially contaminated *L. monocytogenes*. Results determined that the addition of flavoring to the milkshakes significantly lowered both the growth rates and the maximum attained populations of both the naturally and artificially contaminated *L. monocytogenes* during storage at 10°C. Strawberries, along with other berries, are known to have antimicrobial properties (Puupponen-Pimiä et al., 2005; Nohynek et al., 2006). Previous research on strawberry puree and flavorings used in dairy products has determined that there are significant differences in pathogen growth in food products with and without the addition of strawberry (Canganella et al., 1998; Tirloni et al., 2015). For example, a study considering the fate of artificially inoculated *L. monocytogenes* and *E. coli* in strawberry flavored yogurt determined that bacterial loads were lower in the flavored yogurt as compared to that of the plain yogurt during storage at 4°C for 70 days (Tirloni et al., 2015).

Interestingly, *L. monocytogenes* has been reported to reach higher populations in dairy products containing chocolate than in those without (Rosenow and Marth, 1987a,b; Kenney and Beuchat, 2004). One study assessing the differences between growth of artificially inoculated *L. monocytogenes* in different milk products determined that the pathogen actually attained significantly higher maximum populations in chocolate milk than it did in whole or skim milk or cream after 70 days at 4°C storage (Rosenow and Marth, 1987b). Another study looking at the growth kinetics of whole-fat milk, whole-fat chocolate milk, and reduced-fat chocolate milk determined that artificially inoculated *L. monocytogenes* grew to higher population levels in both types of chocolate milk compared to the whole-fat milk during storage at 10°C for 96 h (Kenney and Beuchat, 2004). Although a decrease in both the growth rates and the maximum populations was observed in the current study, the divergence of the results stated here from the published literature may be due to variations in the formulation of the chocolate syrup used for flavoring the milkshakes in this study.

The addition of mint flavoring to the milkshakes in this study had the most significant effect on both naturally and artificially inoculated *L. monocytogenes*, resulting in reduced growth rates, lag phases, and maximum populations compared to the strawberry or chocolate flavorings. Mint has been shown to possess antimicrobial properties against *L. monocytogenes* (Irkin and Korukluoglu, 2009; Lixandru et al., 2010; Akdemir Evrendilek, 2015; Shahbazi, 2015). Only one study in the published literature has assessed the growth dynamics of *L. monocytogenes* in a dairy matrix with mint. Survival and growth of artificially inoculated *L. monocytogenes* was assessed in a yogurt drink in combination with mint and high pressure processing (Akdemir Evrendilek, 2015), and it was shown that the addition of mint to the yogurt drink significantly hindered the growth of *L. monocytogenes*, a conclusion which is also supported in this study.

This is the first report which compares the behavior of *L. monocytogenes* in naturally vs. artificially contaminated food product. In the comparison of both the naturally and artificially contaminated *L. monocytogenes* in the current study, there were significant differences in the growth of both contamination states in milkshakes; significant differences were also seen in the derived secondary models. The most significant difference was observed in the models relating lag phase to temperature (**Figure 4B**). The naturally contaminated *L. monocytogenes* was determined to have much longer lag phases than the artificially contaminated population in milkshakes stored between 5 and 25°C. This difference in lag phases would ultimately determine the risk of this pathogen in milkshakes made from contaminated ice cream. Differences in natural and artificial contamination states should be considered when determining risk for ice cream and milkshakes.

## Author Contributions

JS, DS, and MT conceived and designed the experiments. JS, VB, CC, SN, AS, and DS performed the experiments. JS, VB, and CC analyzed the data. JS wrote the manuscript. CC, DS, and MT critically reviewed the manuscript.

## Conflict of Interest Statement

The authors declare that the research was conducted in the absence of any commercial or financial relationships that could be construed as a potential conflict of interest.

## Footnotes

1 www.combase.cc
